# The prevalence of physical and verbal violence among emergency medicine physicians in military hospitals vs non-military hospitals, Jeddah, Saudi Arabia: multi-center cross-sectional study

**DOI:** 10.1186/s12873-024-01049-z

**Published:** 2024-07-29

**Authors:** Kholoud Abdullah Babkair, Bsaim Abdulsalam Altirkistani, Jamil Mostafa Baljoon, Abdulrahman Adnan Almehmadi, Ahmad Loay Atiah, Sultan Abdullah Alsadan, Montasir Esam Moamena

**Affiliations:** 1 Department of Emergency Medicine, Ministry of the National Guard – Health Affairs, Jeddah, Saudi Arabia; 2 College of Medicine, King Saud bin Abdulaziz University for Health Sciences, Jeddah, Saudi Arabia; 3 King Abdullah International Medical Research Center, Jeddah, Saudi Arabia

**Keywords:** Violence, Emergency Medicine, Military hospitals, Perpetrators, Security, Physician, Workplace violence, Jeddah, Saudi Arabia

## Abstract

**Introduction:**

In healthcare settings, physical and verbal attacks are commonly encountered in the workplace among healthcare providers. Patients and patients’ relatives and friends have been reported to be the perpetrators of workplace violence. Among all healthcare settings, emergency department (ED) have been designated as high-risk settings for violence, where more than one-quarter of emergency physicians reported that they were victims of physical assault. This study aimed to report the prevalence of workplace violence against emergency medicine physicians in military and non-military hospitals in Jeddah city.

**Methodology:**

A cross-sectional design has been used in this study. An electronic questionnaire was developed through the Google Form Platform and it included demographic data, the occurrence of verbal or physical violence in the workplace to participants, how many times they experienced this violence, the time of incidents, the location either inside or outside the hospital, whether the perpetrators were mostly patients, patient families, or friends, and whether they reported any violence or not. Categorical variables were used to describe frequencies and percentages, while descriptive statistics such as mean and 95% Confidence Interval (95% CI) were used to summarize the scale variables. *P* < 0.05 was considered for statistically significant differences.

**Results:**

Among the 100 participants, 76 experienced either physical or verbal violence, or both. The remaining 24 did not experience any sort of violence. 83% of the physicians who have been physically violated were working in non-military hospitals. Of the 72 participants who had experienced verbal violence, 51 (70.8%) were working in a non-military hospital, while 21 (29.2%) were in a military hospital. The most common reason for not reporting was that the participants felt that reporting the violence incidence was useless. Moreover, 92% of participants chose “Train healthcare workers to deal with violent attacks” as a suggested helpful factor in decreasing the number of work-related violence. In addition, “Education of the public” and “Raising awareness of healthcare workers” were chosen as helpful factors as well by 91% and 90% of participants, respectively.

**Conclusion:**

This revealed that physicians in non-military hospitals experience higher levels of violence compared to their military counterparts. However, it is concerning that instances of violence are substantially under-reported across both military and non-military healthcare facilities.

**Supplementary Information:**

The online version contains supplementary material available at 10.1186/s12873-024-01049-z.

## Introduction

Violence is an “intentional use of physical force or power, threatened or actual, against oneself, another person, or against a group or community that either results in or has a high likely-hood of resulting in injury, death, psychological harm, mal-development, or deprivation”, as defined by World Health Organization (WHO) [1]. In healthcare settings, physical and verbal attacks are commonly encountered in workplace among healthcare providers [2–4]. Workplace violence requires public health attention as 44.7% of all healthcare personnel are susceptible to violence every year [5]. Back to the 1980s, the earliest studies that reported workplace violence towards healthcare workers were conducted in the United States where 57% of emergency department (ED) staff were threatened with a weapon [6]. In a recent review, the 12-month prevalence of workplace violence was 70.9% in Australia and New Zealand, 67.3% in North America, 64.9% in Asia, 62.7% in Latin America, 59.2% in Africa, and 48.1% in Europe; therefore, workplace violence has been considered a global problem [7]. Additionally, 91% of general practitioners reported that they have encountered aggression at least once in their career life [8]. Locally, in Saudi Arabia, the prevalence of workplace violence among healthcare providers was 81.4% in Al-Riyadh, 57.5% in Abha, and 48.6% in Arar [9]. Unreported violent acts are also a medical concern as, for example, only 23.8% of healthcare workers who suffered from violence reported the assault incident [10].

Furthermore, patients and patients’ relatives and friends have been reported to be the perpetrators of workplace violence [11–13]. Multiple reasons may contribute to this unpleasant incidence, including not meeting patients’ expectations, waiting for a long time in triage, no pain improvement upon the given medications, and insufficient staff members, either health providers or security guards, all of which may lead to aggressive situations to occur [14, 15]. Suboptimal well-being of healthcare workers may negatively impact their performance and productivity, leading to minimized quality of healthcare delivery [2]. Resolving the issue between healthcare workers and the perpetrator is still unsatisfactory [12]. Among all healthcare settings, EDs have been designated as high-risk settings for violence, where more than one-quarter of emergency physicians reported that they were victims of physical assault [16].

A conducted study in Jordan reported that doctors who are working in governmental hospitals experienced higher incidents of workplace violence, especially during night shifts. While those who are working in military hospitals, in fact, had higher satisfaction rates with workplace violence policies [17]. Furthermore, even though certain cities in Saudi Arabia documented the prevalence of workplace violence among healthcare providers [9, 18], up to our knowledge, no studies compared the prevalence of workplace violence between military and non-military hospitals in Saudi Arabia. Thus, there is a need to address the prevalence of workplace violence in hospitals with insufficient security guarding staff available in the ED in comparison to those hospitals that are considered to be military hospitals.

### Specific objective

The specific objectives of this research were to compare the frequency and percentages of the experienced physical and verbal violence among emergency physicians in military and non-military hospitals, to know the commonest perpetrators of this violence, and to identify factors that might reduce hospital violence.

## Methodology

A cross-sectional design has been used in this study that aims to provide a comprehensive insight into the prevalence of violence against physicians working in emergency departments in military Vs non-military hospitals in Jeddah city. The specific objective of this research to compare the frequency and percentages of the experienced violence between military Vs non-military hospitals among emergency physicians and to know who are the commonest prepetrators in these violences. This study used a convenient sampling technique. The minimum sample size as per reported in Alnofaiey YH et al. [18] as in our study all eligible physicians in the ED of the below defined hospitals (250) were invited to participate in the study, but only 100 agreed to participate. Despite the multiple reminders and several visits to the targeted population of the below mentioned hospitals, the response rate was (40%). This could be contributed to the fact that emergency physicians do not have much time of filling questionnaires while they are in shifts due to the workload encountered by frontline healthcare providers such emergency physicians in which shifts can be unpredictable and busy. Especially as noticed during the data collection that the commonest excuse of refusing to participate was “busy shift, cannot participate”. Therefore, this attributed to result in low response rate; nevertheless, it at least gave insight into the workplace violence among emergency physicians.

An electronic questionnaire was developed through the Google Form Platform. The included hospitals were 8: National Guard Hospital, King Fahad Armed Forces Hospital, King Faisal Specialist and Research Center Hospital, Fakeeh Hospital, Samir Abbas Hospital, three of Minster of Health Hospitals. These centers are known for their workload encountered for serving the community of Jeddah city as well as most of these hospitals are training centers in Jeddah for ED training program, thus they were selected according to their workload, number of emergency physicians, and the provided logistical support for accessing and reaching to the emergency departments to distribute the questionnaire. Fieldwork was from 1 July 2023 till 1 September. The inclusion criteria: emergency medicine physicians on either morning, evening, or night shifts, with various positions ranging from residents to consultants While doctors with different specialties who were rotating in emergency medicine as part of their curricular training program were excluded. The targeted population was reached by visiting the aforementioned hospitals and distributing the questionnaire to eligible physicians as well as reaching out to the chief residents/consultants of emergency medicine and encouraging them to deliver the survey to their emergency medicine trainees. The questionnaire elements consisted of several sections in which they were structured and adopted from previous conducted studies and modified those questions accordingly and added multiple questions as well to match the current study’s goals [9, 14, 18]. The first part was the demographic data such as gender, age-, and work-related information such as training ranks and workplace (military or non-military hospital). The second part was about addressing the main aim of this research which included the occurrence of verbal or physical violence in the workplace to participants. Also, for those who either had verbal or physical violence, how many times they experienced this violence, the time of incidents, the location either inside or outside the hospital, whether the perpetrators were mostly patients, patient families, or friends, and whether they reported any violence or not. The last part was focusing on reflecting participants’ awareness about the process of reporting violence incidents in their hospitals. The questionnaire was sent to several experts in the field of emergency medicine to ensure the validity and clarity of questionnaire’s elements and the suggested modifications were taken into consideration accordingly. Moreover, a pilot study was performed as well by sending the questionnaire to multiple emergency physicians similar to those targeted participants to ensure that all questions are clear and understandable. Then, the data collection started after IRB approval, and the distribution of the questionnaire was performed by the research team via distributing the survey in-person to the targeted participants by visiting military and non-military hospitals during various shift hours as well as through WhatsApp for those who were not available during the shifts. Informed consent from the participants was provided before filling out the questionnaire to ensure the confidentiality and agreement of the participants. The data was kept in secured place and only accessible to research members, and then the data was extracted into Microsoft Excel for data cleaning and analysis. This study was performed in accordance with the Declaration of Helsinki and all relevant guidelines and regulations. This study approved by King Abdullah International Medical Research Centre (KAIMRC), Jeddah, Saudi Arabia. IRB approval study number NRJ22J/290/11. An informed consent was obtained from participants before filling the questionnaire.

### Data analysis

The data analysis was performed using Statistical Software for Social Sciences (SPSS) version 25. Categorical variables were described using frequencies and percentages, while descriptive statistics such as mean and 95% Confidence Interval (95% CI) were used to summarize the scale variables. Chi-Square, Mann-Whitney test and T-Test were used, a significance level of < 0.05 was considered statistically significant, and a confidence level of 95% was used with a margin of error of ± 5%. Mann-Whitney test was done for the age and the number of work violence since Shapiro-Wilk test was significant for both with a *p*-value of 0.0001 and 0.0004, respectively.

## Results

### Demographics

One hundred of emergency medicine physicians agreed to participate in the survey to evaluate the prevalence of physical and verbal violence in military and non-military hospitals in Jeddah, Saudi Arabia. Among the 100 participants, 68 were males and 32 were females. The mean age of participants was 31.89 (95%CI 30.29–33.49) years. Among the 100 participants, 51 were residents, 26 were staff physicians, 9 were registrars, 5 were assistant consultants, and 9 were consultants. Seventy of the one hundred participants were from non-military hospitals, while 30 were from military hospitals. Among the 100 participants, 76 experienced either physical or verbal violence or both. The remaining 24 did not experience any sort of violence. Of the 76 participants who experienced some sort of violence, 4 (5.3%) were only physical, 58 (76.3%) were only verbal, and 14 (18.4%) experienced both physical and verbal violence, as in Table 1.

### Helpful factors to decrease work-related violence

Several helpful factors may decrease the number of work-related violence in which 92% of participants chose “Train healthcare workers to deal with violent attacks” as a suggested helpful factor alongside “Education of the public” and “Raising awareness of healthcare workers” were chosen as well by 91% and 90% of participants, respectively. In contrast, according to the participants, the least effective factors to decrease work-related violence were “Mandatory military training within the training programs of healthcare workers” and “Prevent friends or relatives from accompanying their patient” since only 34% and 60% of the participants agreed that those factors are helpful, respectively.

### Physical violence

Among the 100 participants, 18 had experienced physical violence, all of which occurred in a hospital setting. 15 out of the 18 (83.3%) participants who experienced physical violence were males. Interestingly, 15 out of the 18 (83.3%) of the physicians who had an incidence of physical violence were working in non-military hospitals. Half (9 out of 18) of the incidents occurred by the patient themselves, and 8 of the 18 (44.4%) occurred by a family member. Among the 18 physical violence incidents, 14 (77.8%) were pushed; 8 (44.4%) were grabbed; 5 (27.8%) were pushed to either furniture, equipment, or supplies; and 3 (16.7%) were punched. Of the 18 physical violence incidents, only 7 (38.9%) were reported, while the remaining were not reported. The most common reason for not reporting, which constituted 6 of 11 (54.6%) not reported incidents, was that the participants felt that reporting the violence incidence was useless.

### Verbal violence

Among the 100 participants in this study, 72 experienced at least one incidence of verbal violence in the medical setting. All of the 72 incidences were in hospital. Of the 72 participants who had experienced verbal violence, 47 (65.3%) were males, while 25 (34.7%) were females. Of the 72 participants who had experienced verbal violence, 51 (70.8%) were working in a non-military hospital, while 21 (29.2%) were in a military hospital. The most common number of verbal violence that participants experienced was more than 5 times, which contributed to 29 of 72 (40.3%). Interestingly, 2 of the 72 (2.8%) participants reported that they experience verbal violence every day. Seventy two of participants reported that the most common perpetrator of verbal violence was a family member, with a number of 48 (66.7%). Among the 72 participants who experienced verbal violence, only 14 (19.4%) were reported, while the other 58 incidences were not reported. The most common reason for not reporting was “feeling that the report is useless,” which constituted 44 of 58 (75.9%) of the unreported incidents. Moreover, “do not know to whom they should report” and “being afraid of the negative consequences” were also reasons for not reporting, and they constituted 9 of 58 (15.5%) and 5 of 58 (8.6%), respectively.

### Inferential statistics between participants who experienced a form of violence and participants who did not

**Fig. 1:**
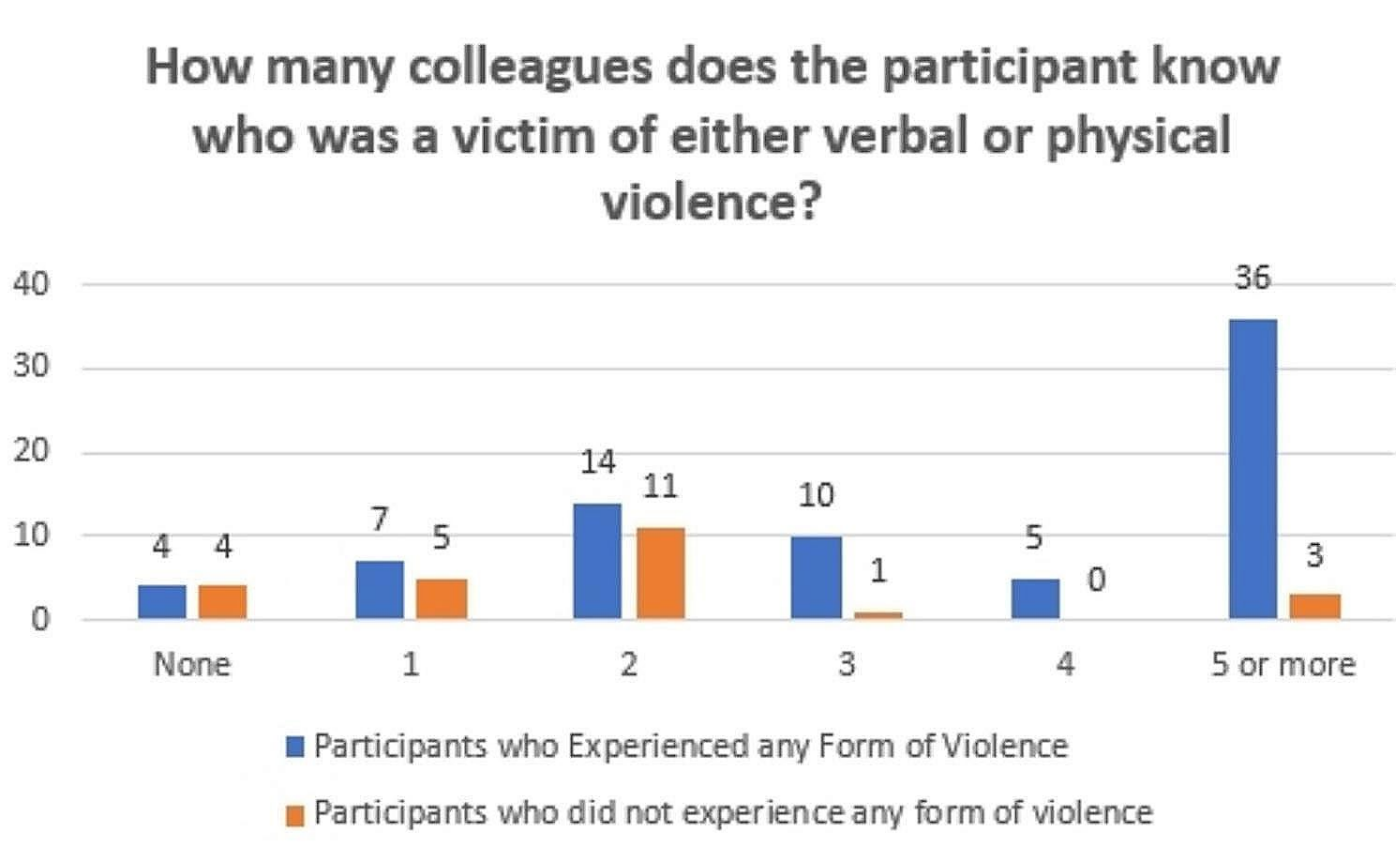
How many colleagues does the participant know who was a victim of either verbal or physical violence? P = 0.001

There was no significant difference between whether the participant experienced a form of violence and the demographic factors, which are: Age, gender, job title, and being in a military hospital. Similarly, there is no significant difference between experiencing a form of violence and knowing the procedure for reporting violence in the hospital since both participants who experienced a form of work-related violence and those who did not have similar results, which constituted almost 40% of both groups do not know the procedure as depicted in Table 2. There is a significant difference between knowing a colleague who is a victim of work violence and experiencing work violence. For instance, 36 of 76 (47.4%) of participants who experienced a form of violence know 5 or more colleagues who were victims of violence. In contrast, only 3 of 24 (12.5%) of participants who did not experience a form of violence know 5 or more colleagues who are victims of violence as illustrated in Fig. 1.

**Table 1:** The demographic characteristics of the participants

Parameter	Value
**Gender (%)**	
Male	68 (68%)
Female	32 (32%)
**Age (95% CI)**	31.89 years old (95% 30.29–33.49).
**Job Title (%)**	
1st year emergency medicine resident	16 (16%)
2nd year emergency medicine resident	10 (10%)
3rd year emergency medicine resident	11 (11%)
4th year emergency medicine resident	14 (14%)
Staff physician	26 (26%)
Registrar	9(9%)
Assistant consultant	5 (5%)
Consultant	9 (9%)
**Type of hospital the participant works in**	
Military Hospital	30 (30%)
Non-Military Hospital	70 (70%)
**Had the participant experience any form of violence in workplace?**	
Yes, verbal violence alone	58 (58%)
Yes, physical violence alone	4 (4%)
Yes, both verbal and physical violence	14 (14%)
No	24 (24%)

**Table 2:** The demographic and inferential statistics difference between participants who experienced any form of violence and participants who did not

Parameter	Participants who Experienced any Form of Violence *N* = 76 (100%)	Participants who did not experience any form of violence *N* = 24 (100%)	*P* value
**Gender (%)**			0.733
Male	51 (67.1%)	17 (70.8%)	
Female	25 (32.9%)	7 (29.2%)	
**Age (95% CI)**	31.41 (29.58–33.24)	33.42 (30.16–36.67)	0.595
**Job title (%)**			0.159
1st year emergency medicine resident	9 (11.8%)	7 (29.2%)	
2nd year emergency medicine resident	8 (10.5%)	2 (8.3%)	
3rd year emergency medicine resident	11 (14.5%)	0 (0%)	
4th year emergency medicine resident	12 (15.8%)	2 (8.3%)	
Staff physician	21 (27.6%)	5 (20.8%)	
Registrar	5 (6.7%)	4 (16.7%)	
Assistant consultant	4 (5.3%)	1 (4.2%)	
Consultant	6 (7.9%)	3 (12.5%)	
**Type of hospital (%)**			0.358
Military Hospital	21 (27.6%)	9 (37.5%)	
Non-Military Hospital	55 (72.4%)	15 (62.5%)	
**Does the participant think they know who is the most common preparatory individual to cause hospital violence? (%)**			0.43
Yes	54 (71.1%)	15 (62.5%)	
No	22 (29%)	9 (37.5%)	
**Does the participant know the procedure for reporting workplace violence in your hospital? (%)**			0.774
Yes	45 (59.2%)	15 (62.5%)	
No	31 (40.8%)	9 (37.5%)	

In this study, ten factors were suggested to decrease work-related violence, only one of which had a significant difference between the group who experienced work-related violence and those who did not. Of the 76 participants who experienced a form of violence, 66 (86.8%) believe that raising the fines against violets is helpful to decrease violence, whereas only 15 of the 24 (62.5%) of the participants who did not experience violence believe that this method is helpful.

In addition, there is no significant difference regarding knowing who the most common preparatory individual is to cause the violence between participants who had work-related violence and those who did not, Table 3.

**Table 3:** The difference between participants who experienced any form of violence and participants who did not regarding the factors that might decrease hospital violence

Parameter	Participants who Experienced any Form of Violence *N* = 76 (100%)	Participants who did not experience any form of violence *N* = 24 (100%)	*P* value
**Preventing friends/relatives to accompany patients is helpful to decrease hospital violence (%)**			0.104
Yes	49 (64.5%)	11 (45.8%)	
No	27 (35.5%)	13 (54.2%)	
**Raising the fines and fees against violets is helpful to decrease hospital violence (%)**			0.008
Yes	66 (86.8%)	15 (62.5%)	
No	10 (13.2%)	9 (37.5%)	
**Establishing police stations in each hospital is helpful to decrease hospital violence (%)**			0.105
Yes	60 (79%)	15 (62.5%)	
No	16 (21.1%)	9 (37.5%)	
**Increasing the number of security guards is helpful to decrease hospital violence (%)**			0.198
Yes	63 (82.9%)	17 (70.8%)	
No	13 (17.1%)	7 (29.2%)	
**Deterrent legislation on the subject is helpful to decrease hospital violence (%)**			0.872
Yes	52 (68.4%)	16 (66.7%)	
No	24 (31.6%)	8 (33.3%)	
**Putting cameras in all areas is helpful to decrease hospital violence (%)**			0.24
Yes	62 (81.6%)	22 (91.7%)	
No	14 (18.4%)	2 (8.3%)	
**Education of the public is helpful to decrease hospital violence (%)**			0.343
Yes	68 (89.5%)	23 (95.8%)	
No	8 (10.5%)	1 (4.2%)	
**Raising awareness of health workers is helpful to decrease hospital violence (%)**			0.755
Yes	68 (89.5%)	22 (91.7%)	
No	8 (10.5%)	2 (8.3%)	
**Train healthcare worker to deal with violence attacks is helpful to decrease hospital violence (%)**			0.427
Yes	69 (90.8%)	23 (95.8%)	
No	7 (9.2%)	1 (4.2%)	
**Mandatory military training within the training programs of healthcare workers is helpful to decrease hospital violence (%)**			0.937
Yes	26 (34.2%)	8 (33.3%)	
No	50 (65.8%)	16 (66.7%)	

### Inferential statistics between the type of violence

In this study, 76 participants experienced some form of violence. The 76 participants were divided into two groups. Group 1 constituted 18 (23.7%) participants who experienced physical violence – either physical violence alone or combined with verbal violence. Group 2 consisted of 58 (76.3%) participants who only experienced verbal violence. There is no significant difference between group 1 and group 2 and the demographic factors – Age, gender, job title, and being in a military hospital. Similarly, there is no significant difference between participants who experienced physical violence and participants who experienced verbal violence only regarding knowing the procedure for reporting incidents in the workplace. Also, there is no significant difference between the two groups regarding knowing who the most common preparatory individual is to cause the violence as in Table 4. Moreover, further details regarding possible factors contribute to decrease violence between participants who experienced verbal violence only vs. participant experience physical violence alone or with verbal violence are shown in Table 5.

**Table 4:** The demographic and inferential statistics difference between participants who experienced verbal violence only and participants who experienced physical violence (alone or with verbal violence)

Parameter	Participants who Experienced verbal violence alone *N* = 58 (100%)	Participants who experienced physical violence *N* = 18 (100%)	*P* value
**Gender (%)**			0.094
Male	36 (62.1%)	15 (83.3%)	
Female	22 (37.9%)	3 (16.7%)	
**Age (95% CI)**	30.31 (28.36–32.26)	34.94 (31.45–38.44)	0.685
**Job title (%)**			0.138
1st year emergency medicine resident	7 (12.1%)	2 (11.1%)	
2nd year emergency medicine resident	7 (12.1%)	1 (5.6%)	
3rd year emergency medicine resident	10 (17.2%)	1 (5.6%)	
4th year emergency medicine resident	11 (19%)	1 (5.6%)	
Staff physician	12 (20.7%)	9 (50%)	
Registrar	3 (5.2%)	2 (11.1%)	
Assistant consultant	4 (6.9%)	0(0%)	
Consultant	4 (6.9%)	2 (11.1%)	
**Type of hospital (%)**			0.234
Military Hospital	18 (31%)	3 (16.7%)	
Non-Military Hospital	40 (69%)	15 (83.3%)	
**Does the participant think they know who is the most common preparatory individual to cause hospital violence? (%)**			0.900
Yes	41(70.7%)	13 (72.2%)	
No	17 (29.3%)	5 (27.8%)	
**Does the participant know the procedure for reporting workplace violence in your hospital? (%)**			0.461
Yes	33 (56.9%)	12 (66.7%)	
No	25 (43.1%)	6 (33.3%)	
**How many colleagues does the participant know who was a victim of either verbal or physical violence? (95% CI)**			0.384
None	1 (1.7%)	3 (16.7%)	
1	6 (10.3%)	1 (5.6%)	
2	11 (19%)	3 (16.7%)	
3	8 (13.8%)	2 (11.1%)	
4	4 (6.9%)	1 (5.6%)	
5 or more	28 (48.3%)	8(44.4%)	

**Table 5:** The difference between participants who experienced verbal violence only and participants who experienced physical violence (alone or with verbal violence)

Parameter	Participants who Experienced verbal violence alone *N* = 58 (100%)	Participants who experienced physical violence *N* = 18 (100%)	*P* value
**Preventing friends/relatives to accompany patients is helpful to decrease hospital violence (%)**			0.432
Yes	36 (62.1%)	15 (83.3%)	
No	22 (37.9%)	3 (16.7%)	
**Raising the fines and fees against violets is helpful to decrease hospital violence (%)**			0.036
Yes	53 (91.4%)	13 (72.2%)	
No	5 (8.6%)	5 (27.8%)	
**Establishing police stations in each hospital is helpful to decrease hospital violence (%)**			0.423
Yes	47 (81%)	13 (72.2%)	
No	11 (19%)	5 (27.8%)	
**Increasing the number of security guards is helpful to decrease hospital violence (%)**			0.955
Yes	48 (83%)	15 (83.3%)	
No	10 (17.2%)	3 (16.7%)	
**Deterrent legislation on the subject is helpful to decrease hospital violence (%)**			0.855
Yes	40 (69%)	12 (66.7%)	
No	18 (31%)	6 (33.3%)	
**Putting cameras in all areas is helpful to decrease hospital violence (%)**			0.826
Yes	47 (81%)	15 (83.3%)	
No	11 (19%)	3 (16.7%)	
**Education of the public is helpful to decrease hospital violence (%)**			0.926
Yes	52 (89.7%)	16 (88.9%)	
No	6 (10.3%)	2 (11.11%)	
**Raising awareness of health workers is helpful to decrease hospital violence (%)**			0.926
Yes	52 (89.7%)	16 (88.9%)	
No	6 (10.3%)	2 (11.11%)	
**Train healthcare worker to deal with violence attacks is helpful to decrease hospital violence (%)**			0.211
Yes	54 (93.1%)	15 (83.3%)	
No	4 (6.9%)	3 (16.7%)	
**Mandatory military training within the training programs of healthcare workers is helpful to decrease hospital violence (%)**			0.937
Yes	20 (34.5%)	6 (33.3%)	
No	38 (65.5%)	12 (66.7%)	

## Discussion

The emergency department is a uniquely stressful environment, with high levels of tension affecting both physicians and patients. This stress, when compounded by external and environmental factors, can potentially lead to instances of verbal or physical violence, posing direct threats to physicians [19–21]. To shed light on this issue, this cross-sectional study aimed to assess the prevalence of physical and verbal violence among emergency medicine physicians in military hospitals and non-military hospitals in Jeddah, Saudi Arabia. The findings revealed a higher incidence of physical violence against doctors in non-military hospitals, which could be attributed to a weaker level of security measures compared to the high level of security in military hospitals. Notably, out of 100 emergency doctors surveyed, 76 reported having experienced physical or verbal violence, or both underscoring the severity of this issue in healthcare settings.

In this study, it was revealed that around 18% of emergency physicians experience physical abuse within hospitals. Astonishingly, it was found that comparable studies in the USA and India reported similar rates of 21% and 17% respectively, while an alarming report from Vancouver cited a physical violence incidence as high as 92%. [22–24]. Common forms of physical violence reported in this study were mainly pushing (77.8%) and grabbing (44.4%), with less frequent instances of punching (16.7%). Half of the physical violence incidents also involved threatening moves or body gestures, while a smaller portion, 11.1%, included attacks with an object. Interestingly, despite the severity of these incidents, only 38.9% were reported to the hospital, leaving a significant proportion unreported. The study also examined the roles and shifts most vulnerable to these incidents. Staff physicians were found to be most at risk, encountering 50% of the physical violence incidents. Evidently, it is revealed that the shift with the highest frequency of physical violence is the evening shift. The perpetrators of these acts were most commonly the patients (50%) and their relatives (44.4%), similar to another study highlighting the same findings [18]. However, a separate study indicated that family relatives were the most common perpetrators of violence against physicians [25].

The study reveals that 72% of emergency physicians experience verbal violence, which is more frequent than physical violence. Similarly, in a study in the USA, the prevalence of verbal violence in emergency healthcare workers is 75% [23]. An even more striking statistic was reported in Vancouver, where the incidence of verbal abuse in the emergency department reached 97% [24]. The role most frequently subjected to verbal violence was the “resident” physician, constituting 54.2% of the reported incidents. Alarmingly, 40.3% of physicians reported experiencing verbal violence more than five times, with 2.8% encountering it daily. The study found that the evening shift, just like physical violence, held the highest frequency of verbal violence. In contrast, another study reported the most physical violence incidents during night shifts, accounting for 61.9% of their results [10]. Predominantly, the perpetrators of verbal violence were family members of the patient (66.7%), followed by the patients themselves (26.4%) and co-workers (6.9%). The most frequently reported forms of verbal violence included loud noises and shouting (93.1%), angry outbursts (87.5%), swearing and cursing (55.6%), and sarcastic, condescending remarks (51.4%). Regrettably, only 19.4% of these incidents were reported, with the main reason for not reporting being a belief that the report would be useless (75.9%). Other reasons included uncertainty about the reporting process (15.5%) and fear of negative consequences (8.6%). Interestingly, a study from Egypt revealed that over 70% of healthcare workers never reported violent incidents to the hospital [10]. This under-reporting of workplace violence by healthcare workers has been widely documented in various literature, emphasizing the need for improved reporting mechanisms and supportive environments in healthcare settings [26, 27].

Out of the 76 participants who encountered violence, they were divided into two groups. Group 1, with 18 participants (23.7%), experienced physical violence, either alone or in combination with verbal violence. Group 2, with 58 participants (76.3%), underwent only verbal violence. No significant differences were found between these two groups in terms of demographics (age, gender, job title, and being in a military hospital) or knowledge of the procedure for reporting workplace incidents. Additionally, both groups had similar awareness levels regarding the most common instigators of violence. However, a key distinction emerged in their attitudes towards increasing the fines/fees against violence: a significantly larger proportion (91.4%) of those who suffered only verbal attacks advocated for increased fines/fees, compared to just 72.2% of those who had experienced physical violence. The Ministry of Health in Saudi Arabia enacted these fines in July 2018 as a deterrent to violence [28].

The study found no significant difference between experiencing violence and demographic factors (age, gender, job title, military hospital status) or knowledge of violence reporting procedures. Meanwhile, another study found that a significant portion of healthcare workers lacked training on the reporting system, leading to their limited awareness and understanding of the formal reporting procedures [28]. The healthcare workers’ solution to reduce workplace violence involved the management actively implementing educational programs to increase the knowledge and education regarding reporting violent incidents and encouraging the physicians to report the incidents more often [29]. Noteworthy, those who experienced violence were significantly more likely to know five or more colleagues who also were victims of violence. Among the ten proposed measures for reducing work-related violence, only the aspect of “increasing fines against offenders” showed a notable difference in approval between individuals who had experienced violence and those who had not. The violence-experienced group exhibited higher support for this measure, with 86.8% in favor, compared to 62.5% in the non-violence-experienced group. However, when evaluating the effectiveness of other proposed measures such as controlling patient accompaniment, establishing police stations, strengthening security personnel, implementing deterrent legislation, installing cameras, public education, health worker awareness programs, violence management training for healthcare workers, and mandatory military training in healthcare worker programs, no significant distinctions were observed between the two groups.

Most of the participants believed that training healthcare workers to manage violent attacks was the most effective way to decrease work-related violence. Likewise, considering public education and increasing awareness among healthcare workers as an effective solution could have a significant impact. As in other studies, training and education revealed that their intervention had a positive effect on staff members’ attitudes and confidence in their ability to deal with and manage situations of violence [30]. Conversely, the least effective strategies, as per the participants, were mandatory military training within healthcare worker programs and preventing friends or relatives from accompanying patients, with only 34% and 60% of the respondents, respectively, considering these factors helpful.

## Conclusion

In conclusion, this research provides a revealing insight into the prevalent issue of violence and abuse inflicted upon medical practitioners, including those in emergency care. The evidence suggests that non-military hospitals experience higher levels of violence compared to their military counterparts. However, it is concerning that instances of violence are substantially under-reported across both military and non-military healthcare facilities. This highlights the significance of training healthcare workers and implementing public education as effective measures to address work-related violence. This underscores the need for acceptable behavior in professional settings. Furthermore, the findings of this study highlight the differences in attitudes towards increasing fines/fees between those who experienced physical violence and those who experienced verbal violence.

### Limitations and recommendations

The main limitation of this study is that the cross-sectional questionnaire relied on self-reported data from emergency medicine physicians, which is subject to recall bias and may not capture the full extent of violence experienced especially as the majority of the included participants were from non-militray hospitals. Also, the small sample size may affect the generalizability of these study’s results.” Furthermore, the study did not assess the long-term psychological impact on physicians who experienced violence, which warrants further investigation. Moreover, the study did not evaluate the effectiveness of the proposed measures in reducing workplace violence, and future research should assess the outcomes of implementing these strategies.

### Electronic supplementary material

Below is the link to the electronic supplementary material.

Supplementary Material 1

## Data Availability

The datasets used and/or analyzed during the current study are available from the corresponding author upon reasonable request.
