# Supplementary material for: The prevalence of physical and verbal violence among emergency medicine physicians in military hospitals vs non-military hospitals, Jeddah, Saudi Arabia: multi-center cross-sectional study
Source: BMC Emerg Med. 2024 Jul 29;24:129. doi: 10.1186/s12873-024-01049-z (PMC11288119; doi:10.1186/s12873-024-01049-z)
Supplement: Supplementary file 1 — Supplementary Material 1 [file 12873_2024_1049_MOESM1_ESM.docx]

Demographic information:

**Gender**

- Male
- Female

**Age in Years:**

**Job title**

- PYG1
- PYG2
- PYG3
- PYG4
- Staff Physician.
- Registrar
- Fellow
- Assistant Consultant
- Consultant
- Other:

**Which hospital do you belong to**

- Non-Military Hospitals
- Military Hospitals

Incidence of Workplace Violence:

1. **Physical Attacks**

**Have you had any physical attack in workplace**

- Yes.*
- No.

*If the answer is**” yes”,** answer the questions with a **star***

**How many times did you experience physical violence? ***

- 1
- 2
- 3
- 4
- More than or equal 5 times
- Other:

**When was the last time you had a physical violence incidence? ***

- Less than 2 Months ago
- Within 2-6 Months ago
- Within 7-12 Months ago
- More than 12 months ago
- Other:

**Where was the location of incident? ***

- Inside the hospital.
- Outside the hospital.

**When was the time of the incident? ***

- Morning shift
- Evening shift
- Night shift
- Outside job/shift hours.

**Who was the perpetrator of the violence? ***

- Patient
- Family of patient
- Friends of patient
- Co-Workers
- I don’t know
- Other:

**Select which of the following actions were involved in the physical violence? ***

Choose **"Yes"** if the option**was**part of incidence

Choose**"No"**if the option **was not**part of incidence

|  | **Yes** | **No** |
| --- | --- | --- |
| Pushing |  |  |
| Punching |  |  |
| Grabbing |  |  |
| Pushing furniture, equipment, and supplies |  |  |
| Threatening moves or body gestures |  |  |
| Attack with an object |  |  |

**Did you report the Incident of Violence? ***

- - - Yes
    - No, I am afraid of negative consequences
    - No, I feel reporting the violence incidence is useless
    - No, I don’t know to whom to report

1. **Verbal Attacks**

**Have you had any Verbal attack in workplace**

- Yes
- No

*If the answer is” yes”, answer the questions with a star*

**How many times did you experience verbal violence? ***

- 1
- 2
- 3
- 4
- More than or equal 5 times
- Other:

**When was the last time you had a verbal violence incidence? ***

- Less than 2 Months ago
- Within 2-6 Months ago
- Within 7-12 Months ago
- More than 12 months ago
- Other:

**Where was the location of incident? ***

- Inside the hospital.
- Outside the hospital.

**When was the time of the incident? ***

- Morning shift
- Evening shift
- Night shift
- Outside job/shift hours.

**Who was the perpetrator of the violence? ***

- Patient
- Family of patient
- Friends of patient
- Co-Workers
- I don’t know
- Other:

**Select which of the following was the type of verbal violence? ***

Choose **"Yes"** if the option**was**part of incidence

Choose**"No"**if the option **was not**part of incidence

|  | **Yes** | **No** |
| --- | --- | --- |
| Loud noises and shouting |  |  |
| Angry outbursts |  |  |
| Swearing and cursing |  |  |
| Sarcastic/condescending comments |  |  |

**Did you report the Incident of Violence? ***

- - - Yes
    - No, I am afraid of negative consequences
    - No, I feel reporting the violence incidence is useless
    - No, I don’t know to whom to report

Interventions

**Do you know the procedure for reporting incidence in the workplace of your hospital**

- Yes
- No

**Which of the following do you think will help much to reduce violence against healthcare workers**

|  | Yes, It is helpful | N, it is NOT helpful |
| --- | --- | --- |
| Prevent friends or relatives to accompany their patient |  |  |
| Raising the fines/fees against those who violets |  |  |
| Establish police stations in each hospital |  |  |
| Increasing number of security guard personnel |  |  |
| Deterrent legislation on the subject |  |  |
| Putting cameras in all areas |  |  |
| Education of the public |  |  |
| Raising awareness of health workers |  |  |
| Train healthcare worker to deal with violence attacks |  |  |
| Mandatory military training within the training programs of healthcare workers |  |  |

**Do you know who is the commonest preparatory individual responsible for physican’s physical / verbal attacks**

- Yes
- No

**If YES, select who is the commonest do you think**

- Patient
- Family of patients
- Friends of patients
- Co-Workers
- Other:……………………

**How many colleague you know who were victims in physical or verbal attack**

- 0
- 1
- 2
- 3
- 4
- More than or equal 5 colleague
